# Howell-Jolly-like inclusions in granulocytes of a liver transplant recipient

**DOI:** 10.1016/j.htct.2025.103978

**Published:** 2025-09-10

**Authors:** Verónica Roldán Galiacho, Sara Hormaza de Jauregui, Lourdes Elicegui Fernández

**Affiliations:** Hematology Department, Hospital Universitario Cruces. 48903 Barakaldo. Bizkaia. Spain

A 38-year-old woman with history of a liver transplant performed four months earlier, presented with fever and multiple lymphadenopathies. She was taking mycophenolate, tacrolimus and prednisone for chronic rejection, lamivudine because of hepatitis B virus serology, and valganciclovir due to recent reactivation of cytomegalovirus.

On admission the complete blood count findings included: hemoglobin 9.7 g/dL, platelets 260 × 10^9^/L, leukocytes 1.5 × 10^9^/L with 0.2 × 10^9^/L neutrophils and elevated C-reactive protein (120 mg/L).

Peripheral blood examination showed hyposegmentation in neutrophils with Howell-Jolly body-like inclusions (Figure 1).

**Figure 1:**
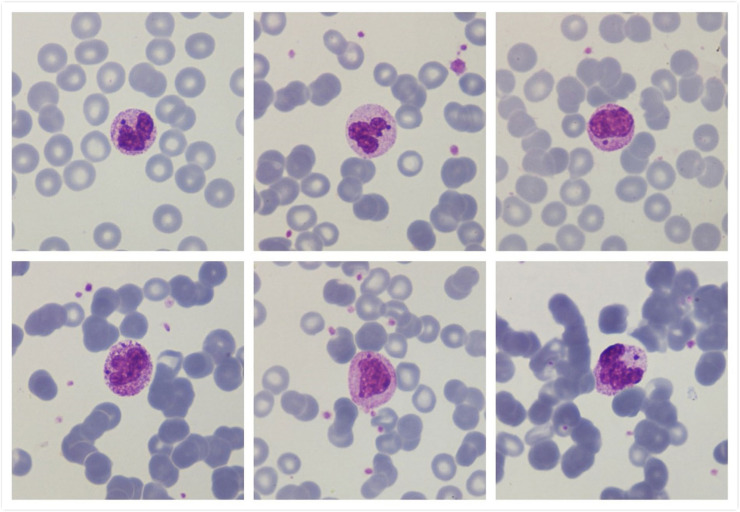
Peripheral blood smear showing atypical inclusions (“Howell-Jollylike-bodies”) in granulocytes (Optical microscopy images using May-Grünwald Giemsa stain - x1000 magnification).

Blood cultures for bacteria and fungus did not support growth of any organism and serologic tests were negative. Additionally, lymph node aspiration cytology did not reveal tumoral cells however, a polymerase chain reaction-based assay to detect *Mycobacterium tuberculosis* in the ganglion was positive. With the diagnosis of ganglionic tuberculosis, the patient received treatment with isoniazid, pyrazinamide, myambutol and levofloxacin. After one year of treatment, the leukocyte count is normal and the adenopathies have disappeared in a full body scan.

Howell-Jolly body-like inclusions in granulocytes are small dense basophilic inclusions similar to Howell-Jolly in erythrocytes. Their appearance in neutrophils may indicate a nuclear fragmentation induced by antiviral treatment with nucleoside analogs, which act on viral DNA. They arise secondary to stressed granulopoiesis often induced by immunosuppressive states including congenital conditions or acquired due to drugs for HIV infection or chemotherapy [1,2]. They are also been described in patients with *Mycobacterium avium* infection and more rarely in myelodysplastic syndromes [3]. These inclusions must be differentiated from other neutrophil inclusions such as those observed in intracellular bacterial infections, those found in genetic conditions such as Chédiak-Higashi syndrome, or Döhle bodies [1].

## Conflicts of interest

The authors of this paper have no conflicts of interest, including specific financial interests, relationships, and/or affiliations relevant to the subject matter or materials included.
